# The Effect and Treatment of PIK3CA Mutations in Breast Cancer: Current Understanding and Future Directions

**DOI:** 10.3390/medicina61030518

**Published:** 2025-03-17

**Authors:** Young-Bin Cho, Kyoung-Sik Park

**Affiliations:** 1 Department of Medicine, Graduate School of Konkuk University, Seoul 05029, Republic of Korea; 2 Department of Surgery, Konkuk University Medical Center, Seoul 05029, Republic of Korea; 3 Department of Surgery, Konkuk University School of Medicine, Seoul 05029, Republic of Korea

**Keywords:** PIK3CA, PIK3CA mutation, PI3K/AKT/mTOR pathway, breast cancer

## Abstract

Gene mutations in PIK3CA, the catalytic subunit of phosphoinositide 3-kinases, are significantly associated with prognosis in breast cancer. This association suggests that breast cancer patients with PIK3CA mutations should receive PIK3CA mutant-specific treatment. This review aimed to investigate novel treatments for PIK3CA-mutant breast cancer. This study investigated the effects of PIK3CA mutations in breast cancer with respect to gene ontology and the PI3K/AKT/mTOR pathway. Subsequently, we comprehensively examined all clinical trials that targeted breast cancer patients with PIK3CA mutations. Finally, this review explored the potential of a new treatment for noncoding RNA.

## 1. Introduction

Breast cancer represents one of the most prevalent causes of cancer-related mortality among females worldwide [1]. A significant proportion of breast cancer patients exhibit genetic mutations. According to cBioportal.gov (invasive breast carcinoma, TCGA, GDC), a public platform for large-scale cancer genomics data sets, breast cancer demonstrates a notable prevalence of gene mutations, particularly in PIK3CA (34%), which constitutes one of the most frequently mutated genes in breast cancer.

PIK3CA serves as the catalytic subunit of PI3Ks, which are phosphoinositide 3-kinases that function as signal transducers in various signaling pathways. The PI3K family is categorized into three classes—I, II, and III, based on their coding genes, distinct structures, and substrate preferences. Class I PI3Ks comprise the catalytic subunit p110 and regulatory subunit p85. The p110 subunit consists of p110α, p110β, and p110δ, encoded by PIK3CA, PIK3CB, and PIK3CD, respectively. The p85 subunit comprises p85α, p85β, and p85γ, which are encoded by PIK3R1, PIK3R2, and PIK3R3, respectively [2]. PIK3CA is located on chromosome 3q26.3, which is 34 kb in length. PIK3CA comprises 20 exons that encode a protein of 1068 amino acids with a molecular mass of 124kDa [3]. Exon 9 encodes the helical domain of the PIK3CA catalytic subunit. E542K and E545K, which are hotspot mutations in exon 9, result in the overactivation of the PIK3CA downstream pathway through the alteration of domains and the disruption of the inhibitory interaction between p110α and p85α [4]. Exon 20 encodes the kinase domains. H1047R, the hotspot mutation in exon 20, reduces the rate of apoptosis in mammary tumors p85α.

Mutations in PIK3CA usually result in poor prognosis in breast cancer. A study of 217 Asian female breast cancer patients demonstrated that PIK3CA mutations were associated with poor prognosis. The relapse-free survival (RFS) rate is significantly shorter in PIK3CA-mutated patients compared to PIK3CA wild-type patients [5]. In human epidermal growth factor receptor 2-positive (HER2+) breast cancer patients undergoing HER2-targeted treatment, patients with PIK3CA mutations showed lower progression-free survival (PFS) than those with wild-type PIK3CA (hazard ratio (HR) 4.602, 95% CI 2.057–10.514, *p* < 0.001) [6]. In hormone receptor-positive/human epidermal growth factor receptor 2-negative (HR+/HER2−) metastatic breast cancer patients, PIK3CA mutations are associated with low overall survival (OS) and PFS [7]. Another study reported that PIK3CA-mutated HR+/HER2-metastatic breast cancer demonstrated inferior overall survival (OS) compared to wild-type PIK3CA (HR 1.44, 95% CI 1.02–2.03) [8]. Patients with wild-type PIK3CA had better OS (HR 0.161, *p* = 0.010) and disease-free survival (DFS) (HR 0.376, *p* = 0.069) than those with PIK3CA mutations in HER2− breast cancer patients. Estrogen receptor-positive (ER+) patients with wild-type PIK3CA had better OS (HR 0.203, *p* = 0.058) compared to those with PIK3CA mutations [9]. These findings suggest that PIK3CA mutations contribute to cell proliferation, apoptosis suppression, and tumorigenesis through the activation of the PI3K/AKT/mTOR pathway.

This review aimed to investigate publications and explore therapeutic approaches for PIK3CA-mutant breast cancer. First, we analyzed PIK3CA mutations in biological mechanisms using public databases and several publications. Second, we systematically reviewed clinical trials targeting PIK3CA mutations in breast cancer. Finally, this study examined noncoding RNAs as potential therapeutic targets for PIK3CA-mutant breast cancer.

## 2. Materials and Methods

### 2.1. Public Platform

STRINGdb, https://string-db.org (accessed on 23 December 2024), is a widely used online portal for Protein–Protein Interaction Networks Functional Enrichment Analysis. We analyzed the proteins that showed significant interactions with PIK3CA proteins using STRINGdb.

GO analysis is a commonly used technique for large-scale functional enrichment research. The proteins which were listed from STRINGdb were characterized according to the functional roles, such as the biological process (BP), cellular component (CC), and molecular function (MF), by the Database for Annotation, Visualization, and Integrated Discovery tools (https://david.ncifcrf.gov/home.jsp) (accessed on 23 December 2024).

### 2.2. Literature Research

PubMed (https://pubmed.ncbi.nlm.nih.gov, firstly accessed on 10 December 2024), was used to search English-language articles related to PIK3CA mutation in breast cancer. The searched articles were published from 1 January 2014 to 31 October 2024, on the keywords PIK3CA and breast cancer.

### 2.3. Search for Clinical Trial

ClinicalTrial.gov (https://clinicaltrials.gov, accessed on 10 December 2024) is a widely used website for clinical trials from around the world. Clinical trials were searched using the following options: breast cancer (condition/disease) and PI3K gene mutation (other terms). Of the above clinical trials, we selected those in which the study status was no longer looking for participants and which was completed.

### 2.4. Statistical Analysis

Gene ontology data were downloaded from the Functional Annotation Chart records in the DAVID tools. Data were analyzed using R statistical software version 4.3.0 (R Foundation for Statistical Computing, Vienna, Austria).

## 3. Results

### 3.1. The Effect of PIK3CA Mutations on the PI3K/AKT/mTOR Pathway

**Figure 1:**
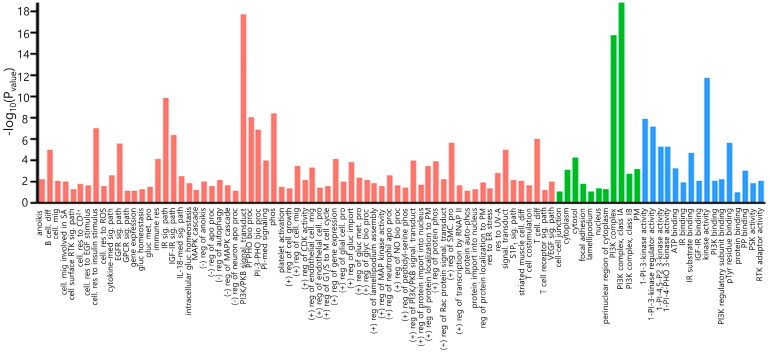
Gene ontology analysis of PIK3CA and proteins interacting with PIK3CA using DAVID (the Database for Annotation, Visualization and Integrated Discovery) tools. Red: biological process; green: cellular component; blue: molecular function. Abbreviations: IL (interleukin); gluc (glucose); EGF (epidermal growth factor); EGFR (epidermal growth factor receptor); PI (phosphatidylinositol); PI3K (phosphatidylinositol 3 kinase); P2 (bisphosphate); IGF-IR (insulin-like growth factor receptor); CDK (cyclin-dependent protein serine/threonine kinase); ER (endoplasmic reticulum); RTK (transmembrane receptor protein tyrosine kinase); pTyr (phosphotyrosine); PHO (phosphotyrosine); S1P1 (sphingosine-1-phosphate receptor); SA (sprouting angiogenesis); PKB (protein kinase B); PSK (protein serine kinase); CD2+ (cadmium ion); ROS (reactive oxygen species); GPCR (G protein-coupled receptor); IR (insulin receptor); glyc (glycogen); VEGF (vascular endothelial growth factor); PM (plasma membrane); RNAP II (RNA polymerase II); PP (protein phosphatase); M (mitosis); NO (nitric oxide); SMC (smooth muscle cell); (−) (negative); (+) (positive); phos (phosphorylation); sig. path (signaling pathway); cell. res (cellular response); cell. mig (cell migration); apo proc (apoptotic process); cell. pro (cell proliferation); cell. diff (cell differentiation); bio proc (biosynthetic process); met. pro (metabolic process); signal. transduct (signal transduction); reg (regulation); res (response); med (mediated).

The association between PIK3CA and the PI3K/AKT/mTOR pathway was analyzed using a public database. STRINGdb (Figure S1) illustrated the direct interaction and potential functional relation among PIK3CA and other proteins: Phosphatidylinositol 3-kinase regulatory subunit beta (PIK3R2), Phosphatidylinositol 3-kinase regulatory subunit gamma (PIK3R3), epidermal growth factor receptor (EGFR), insulin receptor substrate 1 (IRS1), phosphoinositide 3-kinase regulatory subunit 5 (PIK3R5), Phosphatidylinositol 3-kinase regulatory subunit alpha (PIK3RA), GTPase KRas (KRAS), Phosphatidylinositol 4,5-bisphosphate 3-kinase catalytic subunit delta isoform (PIK3CD), RAC-alpha serine/threonine-protein kinase (AKT1), and Phosphatidylinositol 4,5-bisphosphate 3-kinase catalytic subunit beta isoform (PIK3CB). These proteins were categorized into three functional groups in the gene ontology analysis (Figure 1, Table S1): biological process (BP), cellular component (CC), and molecular function (MF). GO analysis showed that BP had the highest regulation of signaling pathways. CC is mainly associated with lamellipodium, cell to cell junction, cytoplasm, and nucleus. MF is primarily associated with protein binding and kinase activity. PI3K signaling plays pivotal roles in various cellular processes, including cell proliferation, transport within the cells, and survival.

**Figure 2:**
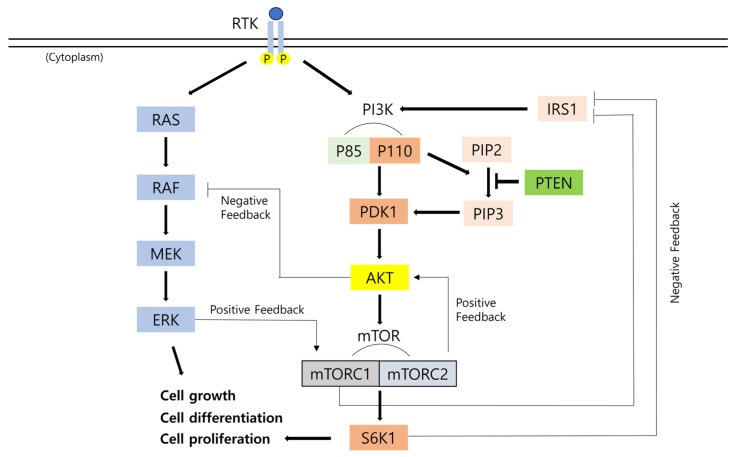
The crosstalk between the PI3K/AKT/mTOR pathway and the MAPK pathway. This figure depicts the feedback loop in signaling pathways.

The PI3K/AKT/mTOR pathway (Figure 2) is initiated by the activation of PI3K through receptor tyrosine kinases (RTKs) [2,10,11]. Activated RTKs bind to the regulatory subunit p85 of PI3K, resulting in the activation of the catalytic subunit p110 of PI3K [12]. Upon the activation of PI3K, phosphatidylinositol 4,5-bisphosphate (PIP2) is converted to phosphatidylinositol 3,4,5-trisphosphate (PIP3) via ATP-induced phosphorylation [13,14]. PIP3 binds to intracellular proteins, serine/threonine protein kinase (AKT), and 3-phosphoinositide-dependent kinase 1 (PDK1). PIP3 functions as a secondary messenger, recruiting AKT and PDK1 to the plasma membrane [12,15]. The hydrophobic pocket of PDK1 induces autophosphorylation and the phosphorylating activation loop T308 of AKT, resulting in AKT activation [16]. Activated AKT translocates from the plasma membrane to the cytoplasm and nucleus, phosphorylating the mammalian target of rapamycin (mTOR), which comprises mTORC1 and mTORC2 [12,17,18]. mTORC1 activates ribosomal S6 kinase 1 (S6K1), thereby inducing cell growth, differentiation, and proliferation. mTORC1 and S6K1 phosphorylate insulin receptor substrate 1 (IRS1), leading to the inactivation or degradation of IRS1. This process creates a negative feedback loop in the PI3K/AKT/mTOR pathway. Conversely, mTORC2 activates AKT, establishing a positive feedback loop [15]. The overactivation of mTOR is significantly associated with human tumors [19]. The PTEN gene, which encodes phosphatase and tensin homolog, regulates the PI3K/AKT pathway by dephosphorylating PIP3 to PIP2. PTEN plays a significant role in maintaining normal cell function and inhibiting aberrant cell proliferation [20,21].

Several studies have indicated that PIK3CA mutations activate the PI3K/AKT/mTOR pathway in breast cancer [12,22]. Cell viability assays, which were conducted with triple-negative breast cancer (TNBC) cell lines harboring PIK3CA hotspot mutations, demonstrated a significant reduction in the proportion of early cell apoptosis in the PIK3CA mutant group compared to the PIK3CA wild-type group (*p* = 0.04) [22]. Furthermore, additional research has revealed that the hotspot mutations of PIK3CA enhance the interaction between p110 and lipid membranes [12]. This interaction results in the dissociation of p110α from both the catalytic core and the p85 regulatory region. The mutations of p110α enhance the membrane binding capacity of p110, thereby activating PI3K signaling [23]. The abnormal activity of the PI3K/AKT/mTOR pathway often induces cellular overgrowth and apoptosis resistance and tumor progression [2].

The PI3K/AKT/mTOR pathway cross-regulates with the MAPK pathway, which comprises a protein cascade that transmits signals from the cell surface to the DNA in the cell nucleus [12,15]. In the MAPK pathway, RTKs activate rat sarcoma virus (RAS), which subsequently recruits and activates rapidly accelerated fibrosarcoma (RAF), mitogen-activated protein kinase kinase (MEK), and extracellular regulated kinase (ERK). Activated AKT inhibits RAF, thereby inducing the negative regulation of the MAPK pathway. Conversely, ERK phosphorylates mTORC1, activating the PI3K/AKT/mTOR pathway. Mutations in either the PI3K/AKT/mTOR pathway or the MAPK pathway may induce the dysregulation of signal transduction and alterations in the feedback loop, potentially leading to tumor development [12].

Several studies have demonstrated that the PI3K signaling pathway plays a significant role in the diagnosis, prognosis, and treatment of breast cancer [12,13,18]. A study analyzed the mutational pattern of the gene in 589 Chinese women. Gene mutations in the PI3K/AKT/mTOR pathway were detected in 62.6% of cases, with the most commonly mutated genes being PIK3CA (45%), PTEN (7.5%), and AKT1 (5.9%) [24]. Consequently, PIK3CA mutations are crucial for PI3K-targeted breast cancer treatment. To evaluate PI3K-targeted treatment, the impact of PIK3CA mutations should be taken into consideration.

### 3.2. Clinical Trials of PI3K Inhibitors in Breast Cancer Patients with PIK3CA Mutations

PI3K inhibitors are categorized into three types: pan-PI3K inhibitors, isoform-specific PI3K inhibitors, and dual PI3K/mTOR inhibitors. Pan-PI3K inhibitors inhibit all isoforms of class PI3K, and alpelisib, pictilisib (GDC-0941), and buparlisib belong to pan-pi3k inhibitors. Isoform-specific PI3K inhibitors target specific isoforms of PI3K. An example of such an inhibitor is alpelisib, which specifically blocks the p110α isoform of PI3K. Similarly, MEN1611 and inavolisib are selective inhibitors targeting the p110α form of PI3K.

Samotolisib and capivasertib are dual PI3K/mTOR inhibitors, targeting both PI3K and mTOR in the PI3K/AKT/mTOR pathway. Table 1 listed clinical trials which were conducted using PI3K inhibitors in PIK3CA-mutant breast cancer according to ClinicalTrial.gov (https://clinicaltrials.gov).

A randomized phase III clinical trial (NCT02340221) demonstrated that taselisib, a pan-PI3K inhibitor, exhibited favorable prognostic outcomes in ER+/HER2− breast cancer. The results indicated that PFS was significantly prolonged in the taselisib group compared to the placebo group (7.4 months vs. 5.4 months, HR = 0.7, *p* = 0.004). The taselisib group showed higher rates of objective response (ORR), clinical benefit (CBR), and duration of objective response compared to the placebo group. Although taselisib had effects on ER+/HER2− breast cancer, this study revealed a high rate of drug discontinuation and dose reduction (36.5%) due to toxicity such as infections, alopecia, pyrexia, and dyspepsia [25]. A randomized study (NCT01437566) investigated the efficacy of pictilisib in ER+ endocrine-resistant breast cancer. The findings indicated that median progression-free survival (mPFS) was higher in the pictilisib group compared to the placebo group for both PIK3CA wild-type and mutant type; however, the difference was not statistical significance. In addition, gastrointestinal and skin toxicities were more observed in the pictilisib group than in the placebo group [26]. In the phase 1b clinical trial (NCT01219699), postmenopausal ER+ breast cancer patients received alpelisib in combination with fulvestrant following antiestrogen therapy. The patients were stratified into two subgroups based on their tumor-tissue PIK3CA mutation status. The ORR of the PIK3CA mutant group was 29% (95% CI 17–43%), while the PIK3CA wild-type group had no overall response. The mPFS was observed to be higher in the PIK3CA mutant group (9.1 months, 95% CI 6.6–14.6) compared to the PIK3CA wild-type group (4.7 months, 95% CI 1.9–5.6). No dose-limiting toxicities (DLTs) were reported with alpelisib administered at doses ranging from 300 mg to 350 mg, whereas 10% of patients who received 400 mg of alpelisib exhibited DLTs, including diarrhea and vomiting. Furthermore, 11% of patients who received 400 mg of alpelisib in combination with fulvestrant experienced DLTs such as hyperglycemia and rash [28]. In the phase I/II study (NCT02379247), patients with HER2− metastatic breast cancer received alpelisib in combination with nab-paclitaxel following chemotherapy. The study population was stratified into two groups based on PIK3CA mutation status: mutated or wild-type. PFS was higher in the PIK3CA mutation group than in the PIK3CA wild-type group (PFS: 11.9 vs. 7.5 months; HR = 0.44, *p* = 0.027). While the common adverse events were diarrhea, hyperglycemia, and rash, there were no patients who discontinued treatment due to those adverse events [29]. In the SOLAR-1 phase II study (NCT03056755), patients with HR+/HER2− advanced breast cancer harboring PIK3CA mutations received alpelisib plus fulvestrant following CD4/6 inhibitor treatment with the aromatase inhibitor [30]. In the primary endpoint, 50.4% of participants exhibited PFS at 6 months (95% CI 41.2–59.6). The ORR was 17% (95% CI 11–25), and primary results showed that CBR was 45% (95% CI 36–55). Twenty-one percent of patients discontinued the clinical trial due to adverse effects, with rash being the most prevalent. No treatment-related fatalities were reported. The SOLAR-1 trial (NCT02437318) demonstrated that the long-term administration of alpelisib plus fulvestrant in HR+/HER2− breast cancer patients with PI3KCA- mutations contributes to improved overall survival (OS). Furthermore, the alpelisib group exhibited a lower rate of treatment discontinuation compared to the placebo group [31]. In the phase 1b clinical trial (NCT03767335), HER2+ advanced breast cancer patients with the PIK3CA mutant received MEN1611 plus only trastuzumab (Group A) or MEN1611 plus trastuzumab and fulvestrant (Group B). The proportion of stable disease was similar between the two groups, while the partial response rates were 36% (Group A) and 28% (Group B), respectively. In addition, 20% of all patients, regardless of fulvestrant administration, experienced treatment-emergent adverse events, with the most frequent being diarrhea (64.3%), nausea (42.8%), and asthenia (31%). While most of the adverse events were reversible and manageable, some adverse events caused treatment interruption (33.3%) and dose reduction (16.7%), primarily due to hyperglycemia, diarrhea, nausea, and asthenia [32]. In the phase Ib study (NCT02124148), nine of the TNBC patients with the PIK3CA mutation received samotolisib plus prexasertib, resulting in an ORR of 13.3%. During dose escalation, DLTs were not observed; however, dose reduction toxicities were noted due to leukopenia, neutropenia, thrombocytopenia, and nausea (52.8%) [33]. A double-blind randomized study (NCT04191499) evaluated the efficacy and safety of inavolisib in combination with palbociclib and fulvestrant in HR+/HER2− advanced breast cancer. The results demonstrated that mPFS was significantly longer in the inavolisib group than in the placebo group (15 vs. 7.3 months). The ORR of the inavolisib group was 58.4%, whereas the placebo group exhibited an ORR of 25.0%. The inavolisib group showed a higher discontinuation rate due to adverse events compared to the placebo group (6.8% vs. 0.6%), while disease progression or death was lower (HR 0.43; *p* < 0.001) [34]. In a randomized phase III clinical trial (NCT04305496), capivasertib in combination with fulvestrant demonstrated efficacy in the treatment of HR+/HER2− metastatic breast cancer patients. However, discontinuation due to adverse events occurred in 13% of the capivasertib group, compared to 2% in the placebo group [35]. Although capivasertib was not a PI3K inhibitor, it was approved for PIK3CA-mutant patients.

The above clinical trials suggested that PI3K inhibitors had an effect on the prognosis of PIK3CA mutations in breast cancer patients. The most prevalent side effects were gastrointestinal in nature, such as diarrhea and nausea, as well as hyperglycemia and cutaneous eruptions.

### 3.3. Future Directions: Noncoding RNAs Targeting Gene Mutations

Noncoding RNAs (ncRNAs) have emerged as potential molecular-level therapeutic agents for various diseases, including cancer, due to their ability to regulate the expression of genes associated with cell cycle or proliferation [36].

MicroRNAs (miRNAs) are small regulatory ncRNA molecules, typically comprising approximately 22 nucleotides. miRNAs bind to the 3 UTRs of target mRNAs, resulting in either the degradation of mRNAs or the repression of target gene translation [37]. It suggests that miRNAs can assume dual roles as either tumor suppressors or oncogenes, contingent upon their specific target genes. In their capacity as tumor suppressors, miRNAs inhibit the expression of oncogenes and impede uncontrolled cell growth and division [38,39]. For example, miR-760 targets the HM13 gene which performs oncogenic function by activating the PI3K/AKT/mTOR pathway and promoting cell proliferation in breast cancer [40]. miR-944 regulates the PI3K/AKT/mTOR pathway by inhibiting SPP1. It was suggested that the downregulation of miR-944 facilitates the expression of SPP1, which subsequently stimulates the PI3K/AKT/mTOR pathway in in vitro experiments with breast cancer cells. In vivo experiments confirmed that miR-944 plays the anticancer role, suppressing SPP1 and the PI3K/AKT/mTOR pathway in breast cancer [41]. miR-203a can also act as a tumor suppressor through directly interacting with PIK3CA. This interaction controls the activation level of the PI3K/AKT/mTOR pathway and can be overactivated by PIK3CA mutations [42]. Conversely, some miRNAs can function as oncogenes by activating the PI3K/AKT/mTOR pathway. miR193 inhibits growth family member 5 (ING5), which suppresses the PI3K/AKT/mTOR pathway. miR193 binds to the 3 UTRs of ING5, thereby promoting cell growth and invasion in breast cancer [43,44,45].

In addition, miRNAs could induce drug resistance breast cancer. miR-205 plays a crucial role in modulating resistance mechanisms in breast cancer through its influence on epithelial–mesenchymal transition (EMT), cancer stem cells properties, and treatment responses. Specifically, miR-205 inhibits EMT by targeting transcriptional repressors, consequently leading to increased invasiveness and resistance to therapeutic interventions [46]. The downregulation of miR-205 contributes to therapy resistance as it leads to enhanced EMT and the maintenance of cancer stem cell properties [46,47]. Furthermore, the interaction between miRNAs and PI3K inhibitors could regulate key oncogenic pathways in the PI3K/AKT/mTOR pathway and improve targeted cancer therapies. For instance, miR-1287-5p can interact with PI3K inhibitors. miR-1287-5p directly targets the PIK3CB gene, which encodes the p110β catalytic subunit of PI3K, thereby downregulating the PI3K/AKT/mTOR pathway. This interaction leads to reduced proliferation and the increased apoptosis of TNBC cells. Also, the overexpression of miR-1287-5p sensitizes TNBC cells to PI3K inhibitors, suggesting a potential therapeutic strategy that combines miR-1287-5p modulation with PI3K inhibition to enhance anticancer efficacy [48].

Long noncoding RNAs (lncRNAs) are heterogeneous ncRNAs exceeding 200 nucleotides in length. lncRNAs facilitate gene regulation, functioning as scaffolds for protein complexes, sequestering miRNAs, or engaging in direct interactions with chromatin to modulate gene expression. lncRNAs have been extensively implicated in the progression and development of cancer as either oncogenes or tumor suppressors [36]. For example, LINC01133 stimulated mTORC2, leading to the activation of AKT and cell proliferation in TNBC [49]. However, GAS5 exerts its tumor-suppressive effects by interacting with the PI3K/AKT/mTOR pathway through the upregulation of PTEN and the direct inhibition of AKT activation. It suggests that GAS5 possesses potential as a therapeutic target in breast cancer treatment [50]. Furthermore, lnRNA H19 plays a critical role in mediating drug resistance in breast cancer through the modulation of drug efflux, apoptosis, and miRNA interactions [51,52]. H19 may influence the expression of tumor-suppressive miRNAs and drug efflux transporters, resulting in drug resistance and decreased intracellular concentrations of chemotherapeutic agents. In addition, H19 has been implicated in the inhibition of apoptosis, enabling cancer cells to survive despite chemotherapy-induced DNA damage [51]. In addition, DUXAP8 exhibited overexpression in breast cancer and demonstrated association with poor prognosis, including radiation resistance [53]. It suggested that lncRNAs may serve as potential therapeutic targets in PIK3CA mutant breast cancer.

Circular RNAs (circRNAs) constitute a distinct category of ncRNAs due to their structural characteristics. circRNAs are formed as closed loops of single-stranded RNAs derived from pre-mRNAs, wherein the 5’ terminus is covalently linked to the 3’ terminus [54]. circRNAs exhibit remarkable stability, potentially impeding interactions with target mRNAs. A study [55] demonstrated that circ-ARHGER28 could serve as a potential diagnostic marker of breast cancer (AUC = 0.889, 95% CI 0.780–0.998, *p* < 0.01), resulting from a lower expression level in breast cancer tissue than in paracarcinoma tissue. Furthermore, circ-ARHGER28 suppresses mRNA expressions of PI3K, AKT, and mTOR. circRNAs function as effective miRNA sponges. For instance, circTFF1 sponges miR-326, subsequently promoting the progression of breast cancer. An in vitro study [56] showed that MCF-7 cells, knockdowns of circ-TFF1, decreased cell migration, proliferation, and invasion.

These studies demonstrated that ncRNAs exhibit significant roles for PIK3CA mutant breast cancer by modulating the PI3K/AKT/mTOR pathway and inhibiting tumorigenicity. They suggested that ncRNAs may serve as potential therapeutic targets or agents for PIK3CA mutant breast cancer by modulating the expression levels of PIK3CA genes. However, further research is necessary to facilitate the clinical application of ncRNAs.

## 4. Discussion

There are several challenges to be solved in cancer treatment, primarily tumor heterogeneity and drug resistance. Tumor heterogeneity in breast cancer significantly influences treatment response and contributes to drug resistance. Breast cancer represents a heterogeneous entity, encompassing diverse subtypes such as HR+/HER2− and TNBC, each characterized by distinct clinical features and responses to therapeutic interventions [36]. The intra-tumoral heterogeneity of tumors encompasses genetic, epigenetic, and microenvironmental heterogeneity. Genetic heterogeneity contributes to drug resistance through the prevention of drug–target binding site interactions, the activation of alternative pathways, or the degradation of regulators. Epigenetic heterogeneity induces resistance phenotypes such as the drug efflux pump. It also promotes cancer stem cell phenotypes through the inhibition of cell cycle regulation, the activation of the cell survival pathway, and the evasion of cell death. Microenvironmental heterogeneity impairs the delivery of therapeutic agents due to the dense extracellular matrix and abnormal vasculature. It also induces cell survival signaling through ECM attachment and inflammatory cytokines. Therefore, the intra-tumoral heterogeneity of PIK3CA-mutant tumors may exhibit varying PIK3CA mutations, resulting in differential responses to PI3K inhibitors [57].

Furthermore, the PI3K/AKT/mTOR pathway involves multiple feedback loops and crosstalk nodes with other signal transduction axes. The dysregulation of the PI3K/AKT/mTOR pathway contributes substantially to the development of cancer drug resistance [11,12]. Resistance to PI3K inhibitors is frequently mediated by the adaptive activation of the MAPK pathway, primarily through feedback activation of RTKs, the loss of negative regulation, and ERK-driven transcriptional adaptation. Despite the inhibition of PI3K, a reduction in activated S6K1 leads to increased RTK signaling by the loss of S6K1-mediated negative feedback on IRS1. RTK activation enhances MAPK signaling, promoting cell proliferation and survival [11]. In the development of inhibitors targeting the PI3K/AKT/mTOR pathway, it is essential to consider dysregulation by mutations and additional activation, as these factors may contribute to drug resistance. Although the PI3K/AKT/mTOR and MAPK pathways have distinct mechanisms, they share numerous downstream targets that can potentially promote cell proliferation and facilitate drug resistance [58]. To address those challenges in cancer treatment, combination strategies, such as combining PI3K inhibitors with MEK inhibitors for co-targeting the PI3K and MAPK pathways, may enhance efficacy [58,59]. A study indicated that the dual downstream blockade of the MAPK and PI3K/AKT/mTOR pathways was more effective in a therapeutic strategy for pancreatic cancer [59]. In an in vitro study, non-small-cell lung cancer (NSCLC) cells with PIK3CA mutations induced more effect responses of anti-proliferation and pro-apoptosis through the combined treatment of a dual PI3K/mTOR inhibitor and MEK inhibitor, compared to exclusive use [60].

Molecular profiling has potential to elucidate cancer heterogeneity and drug resistance in breast cancer [61,62]. A study of cancer genomes with PIK3CA mutations revealed that approximately 15% of breast cancers exhibit multiple PIK3CA mutations, with 95% being double mutations. These double mutations result in enhanced PI3K downstream signaling, cell proliferation, and tumor growth [63]. In addition, the PIK3CA-mutant tumor might respond well to the selective inhibition of PIK3CA, such as copanlisib or alpelisib, rather than PI3K inhibitors, since the activation event is selective [64]. Drug resistance in TNBC cells is facilitated by a coordinated reprogramming of MAPK pathway inhibition. The administration of a multi-targeted tyrosine kinase inhibitor against the upregulated kinases can restore an efficacious drug response in these cells. It suggests that identifying and inhibiting the adaptive response mechanisms of cancer cells can lead to enhanced anti-tumor efficacy [62].

This review analyzed the impact of PIK3CA mutations on breast cancer, a disease that has long affected women worldwide. This study comprehensively examined all clinical trials involving breast cancer patients with PIK3CA mutations. Furthermore, we investigated the potential of ncRNAs as anticancer agents in PIK3CA-mutant breast cancer.

## 5. Conclusions

Breast cancer exhibits PIK3CA mutations at a frequency of approximately 34%. PIK3CA exerts clinical effects on patients with breast cancer, particularly through the PI3K/AKT/mTOR pathway. The aberrant activity of the PI3K/AKT/mTOR pathway frequently promotes excessive cell division and resistance to apoptosis, contributing to the development and progression of tumors. Several clinical trials have been conducted on PIK3CA-mutant breast cancer. Breast cancer patients with PIK3CA mutations demonstrated distinct outcomes compared to PIK3CA wild-type patients. These results suggest that breast cancer treatment should be conducted based on patients’ molecular subtypes in the future.

## Figures and Tables

**Table 1:** List of clinical trials that targeted PIK3CA mutations in breast cancer.

NCT (Phase)	Patient Population	Treatment	Clinical Outcomes	Adverse Effects	Reference
NCT02340221 (III)	ER+/HER2− ABC or mBC with PIK3CA-mutant	taselisib + fulvestrant / placebo + fulvestrant	Taselisib group vs. placebo group PFS: 7.4 vs. 5.4; HR 0.70, *p* = 0.0037 ORR: 28.0% vs. 11.9%, 95% CI 8.4–23.8	diarrhea, hyperglycemia, nausea, fatigue, and rash	[25]
NCT01437566 (II)	ER+/HER2− BC	pictilisib + fulvestrant / placebo + fulvestrant	Pictilisib group vs. placebo group mPFS: 6.6 vs. 5.1; HR = 0.74, *p* = 0.096 mPFS of PIK3CA mutation: 6.5 vs. 5.1; HR = 0.73, *p* = 0.268 mPFS of PIK3CA WT: 5.8 vs. 3.6; HR = 0.72, *p* = 0.23 ORR: 7.3% vs. 5%; *p* = 0.73 CBR: 19.5% vs. 35%; *p* = 0.19	hyperglycemia, pneumonitis, diarrhea, and rash	[26]
NCT01339442 (I)	ER+ mBC	buparlisib + fulvestrant	No significant response or duration of treatment in PIK3CA mutant group	fatigue, hyperglycemia, and rash	[27]
NCT01219699 (Ib)	ER+ BC	alpelisib + fulvestrant	PIK3CA-altered vs. PIK3CA wild-type ORR: 29% vs. 0% mPFS: 9.1 [95% CI 6.6–14.6] vs. 4.7 [95% CI 1.9–5.6]	diarrhea, nausea, hyperglycemia, and rash	[28]
NCT02379247 (I/II)	HER2− mBC	alpelisib + nab-paclitaxel	PIK3CA-altered vs. PIK3CA wild-type PFS: 11.9 vs. 7.5; HR = 0.44, *p* = 0.027 CBR: 100% vs. 68%; OR = 1.47, *p* = 0.013 mOS: 26.7 vs. 14.9; HR =0.59, *p* = 0.19	hyperglycemia, neutropenia, diarrhea, and rash	[29]
NCT03056755 (II)	HR+/HER2- ABC with PIK3CA-mutant	alpelisib + fulvestrant	In the primary endpoint, 6-month PFS: 54%, 95% CI 44–63 PFS: 7.3 months, 95% CI 5.6–8.3 mOS: 17.3 months, 95% CI 17.2–20.7 ORR: 17%; 95% CI 11–25 CBR: 45%, 95% CI 36–55	rash, hyperglycemia, and diarrhea	[30]
NCT02437318 (III)	HR+/HER2- ABC with PIK3CA-mutant	alpelisib + fulvestrant / placebo+ fulvestrant	Alpelisib group vs. placebo group mOS: 39.3 vs. 31.4; HR = 0.86, *p* = 0.15 Disease progression: 65.7% vs. 80.2%	rash, hyperglycemia	[31]
NCT03767335 (Ib)	HER2+ ABC With PIK3CA-mutant	MEN1611 + trastuzumab / MEN1611 + trastuzumab + fulvestrant	MEN1611 + trastuzumab vs. MEN1611 + Trastuzumab + Fulvestrant PR: 36% vs. 28% Stable disease: 55% vs. 56%	diarrhea, nausea, asthenia, anemia, and hyperglycemia	[32]
NCT02124148 (Ib)	mTNBC	samotolisib + prexasertib	In advanced or metastatic cancer with hotspot PIK3CA mutation, lobular breast carcinoma showed ORR of 13.3%.	vomiting, rash	[33]
NCT04191499 (III)	HR+/HER2− mBC With PIK3CA-mutant	inavolisib + palbociclib + fulvestrant / placebo + palbociclib+ fulvestrant	Inavolisib group vs. placebo group PFS: 15 vs. 7.3 ORR: 58.4% vs. 25.0% Disease progression or death: HR = 0.43, *p* < 0.001	neutropenia, hyperglycemia, rash, and diarrhea	[34]
NCT04305496 (III)	HR+/HER2− mBC	capivasertib + fulvestrant / placebo + fulvestrant	Capivasertib group vs. placebo group PFS: 7.3 vs. 3.1; HR = 0.5, *p* < 0.001 Time to deterioration: 24.0 vs. 12.0; HR = 0.7, 95% CI 0.53–0.92	rash, diarrhea, nausea, and hyperglycemia	[35]

HER2: human epidermal cell receptor 2, ER: estrogen receptor, BC: breast cancer, ABC: advanced breast cancer, mBC: metastatic breast cancer, ORR: objective response rate, mPFS: median progression-free survival, mOS: median overall survival, CBR: clinical benefit rate, PR: partial response, HR: hazard ratio, and WT: wild-type. Survival unit: months.

## Supplementary Materials

The following supporting information can be downloaded at: https://www.mdpi.com/article/10.3390/medicina61030518/s1, Figure S1: STRINGdb for predicting proteins interacting with PIK3CA; Table S1: Gene ontology analysis using DAVID tools.
