# Supplementary material for: The Effect and Treatment of PIK3CA Mutations in Breast Cancer: Current Understanding and Future Directions
Source: Medicina (Kaunas). 2025 Mar 17;61(3):518. doi: 10.3390/medicina61030518 (PMC11944057; doi:10.3390/medicina61030518)
Supplement: Supplementary file 1 [file medicina-61-00518-s001.zip › Table S1.pdf]

Table S1. Gene ontology analysis using DAVID tools.

	Category	Pathway/Activity	$-log_{10}(P_{value})$
1	BP	anoikis	2.217123194
2	BP	B cell differentiation	4.968347513
3	BP	cell migration	2.074103244
4	BP	cell migration involved in sprouting angiogenesis	1.974976027
5	BP	cell surface receptor protein tyrosine kinase signaling pathway	1.260762329
6	BP	cellular response to cadmium ion	1.754512416
7	BP	cellular response to epidermal growth factor stimulus	1.646356826
8	BP	cellular response to insulin stimulus	7.003593015
9	BP	cellular response to reactive oxygen species	1.576084270
10	BP	cytokine-mediated signaling pathway	2.577311021
11	BP	epidermal growth factor receptor signaling pathway	5.568217179
12	BP	G protein-coupled receptor signaling pathway	1.099241915
13	BP	gene expression	1.159821847
14	BP	glucose homeostasis	1.249476195
15	BP	glucose metabolic process	1.488632474
16	BP	immune response	4.103883018
17	BP	insulin receptor signaling pathway	9.886727341
18	BP	insulin-like growth factor receptor signaling pathway	6.336117829
19	BP	interleukin-18-mediated signaling pathway	2.517559080
20	BP	intracellular glucose homeostasis	1.835588933
21	BP	MAPK cascade	1.207193072
22	BP	negative regulation of anoikis	2.018243777
23	BP	negative regulation of apoptotic process	1.587374874
24	BP	negative regulation of macroautophagy	2.120510180
25	BP	negative regulation of MAPK cascade	1.636910373
26	BP	negative regulation of neuron apoptotic process	1.131048369
27	BP	phosphatidylinositol 3-kinase/protein kinase B signal transduction	17.73537443
28	BP	phosphatidylinositol phosphate biosynthetic process	8.050845529
29	BP	phosphatidylinositol-3-phosphate biosynthetic process	6.842922446
30	BP	phosphatidylinositol-mediated signaling	4.000963878
31	BP	phosphorylation	8.428166927
32	BP	platelet activation	1.502007708
33	BP	positive regulation of cell growth	1.359334024
34	BP	positive regulation of cell migration	3.470233088
35	BP	positive regulation of cyclin-dependent protein serine/threonine kinase activity	2.150374408

36	BP	positive regulation of endothelial cell migration	3.312253050
37	BP	positive regulation of endothelial cell proliferation	1.444904705
38	BP	positive regulation of G1/S transition of mitotic cell cycle	1.576084270
39	BP	positive regulation of gene expression	4.090566209
40	BP	positive regulation of glial cell proliferation	1.974976027
41	BP	positive regulation of glucose import	3.816800816
42	BP	positive regulation of glucose metabolic process	2.341764895
43	BP	positive regulation of glycogen biosynthetic process	2.120510180
44	BP	positive regulation of lamellipodium assembly	1.820964608
45	BP	positive regulation of MAP kinase activity	1.560195107
46	BP	positive regulation of neutrophil apoptotic process	2.596641293
47	BP	positive regulation of nitric oxide biosynthetic process	1.656017794
48	BP	positive regulation of peptidyl-serine phosphorylation	1.439013092
49	BP	positive regulation of phosphatidylinositol 3-kinase/protein kinase B signal transduction	3.985199656
50	BP	positive regulation of protein import into nucleus	1.676023430
51	BP	positive regulation of protein localization to plasma membrane	3.439590600
52	BP	positive regulation of protein phosphorylation	3.894661785
53	BP	positive regulation of Rac protein signal transduction	2.217123194
54	BP	positive regulation of smooth muscle cell proliferation	5.639622652
55	BP	positive regulation of transcription by RNA polymerase II	1.657291451
56	BP	protein autophosphorylation	1.136642211
57	BP	protein import into nucleus	1.276307151
58	BP	regulation of protein localization to plasma membrane	1.935665413
59	BP	response to endoplasmic reticulum stress	1.322334802
60	BP	response to UV-A	2.818291964
61	BP	signal transduction	4.989031955
62	BP	sphingosine-1-phosphate receptor signaling pathway	2.120510180
63	BP	striated muscle cell differentiation	2.092580447
64	BP	T cell costimulation	1.665903142
65	BP	T cell differentiation	6.004883230
66	BP	T cell receptor signaling pathway	1.220809665
67	BP	vascular endothelial growth factor signaling pathway	2.018243777
68	CC	cell-cell junction	1.052216961
69	CC	cytoplasm	3.070682454
70	CC	cytosol	4.234137694
71	CC	focal adhesion	1.758602158
72	CC	lamellipodium	1.047925355

73	CC	nucleus	1.354780023
74	CC	perinuclear region of cytoplasm	1.308598020
75	CC	phosphatidylinositol 3-kinase complex	15.73671425
76	CC	phosphatidylinositol 3-kinase complex, class IA	18.81536302
77	CC	phosphatidylinositol 3-kinase complex, class IB	2.718096807
78	CC	plasma membrane	3.162111769
79	MF	1-phosphatidylinositol-3-kinase activity	7.921187777
80	MF	1-phosphatidylinositol-3-kinase regulator activity	7.168687330
81	MF	1-phosphatidylinositol-4,5-bisphosphate 3-kinase activity	5.295410402
82	MF	1-phosphatidylinositol-4-phosphate 3-kinase activity	5.295410402
83	MF	ATP binding	3.238818014
84	MF	insulin receptor binding	1.945350093
85	MF	insulin receptor substrate binding	4.726255217
86	MF	insulin-like growth factor receptor binding	2.083045713
87	MF	kinase activity	11.77937320
88	MF	phosphatidylinositol 3-kinase binding	2.083045713
89	MF	phosphatidylinositol 3-kinase regulatory subunit binding	2.245266991
90	MF	phosphotyrosine residue binding	5.611049220
91	MF	protein binding	1.012412351
92	MF	protein phosphatase binding	3.034630224
93	MF	protein serine kinase activity	1.836000763
94	MF	transmembrane receptor protein tyrosine kinase adaptor activity	2.056817965

* BP: Biological Process. CC: Cellular Component. MF: Molecular Function.
