# Supplementary figures and images for: The Effect and Treatment of PIK3CA Mutations in Breast Cancer: Current Understanding and Future Directions
Source: Medicina (Kaunas). 2025 Mar 17;61(3):518. doi: 10.3390/medicina61030518 (PMC11944057; doi:10.3390/medicina61030518)

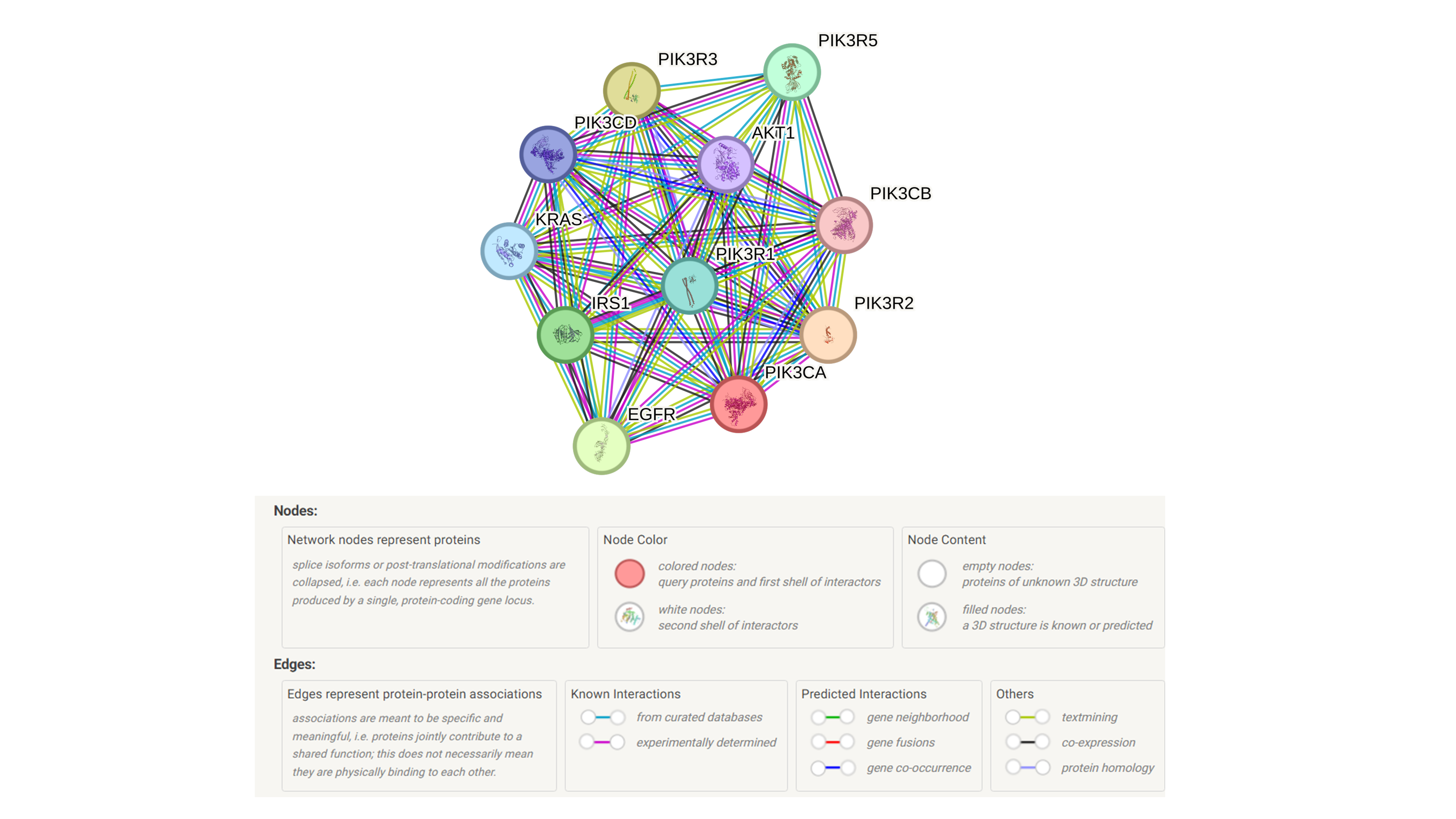

Supplement: Supplementary file 1 [file medicina-61-00518-s001.zip › Figure S1.tif]
